# Impact of iron raw materials and their impurities on CHO metabolism and recombinant protein product quality

**DOI:** 10.1002/btpr.3148

**Published:** 2021-05-03

**Authors:** Christine H. Weiss, Corinna Merkel, Aline Zimmer

**Affiliations:** ^1^ Merck Life Science, Upstream R&D Darmstadt Germany; ^2^ Institute for Organic Chemistry and Biochemistry, Technische Universität Darmstadt Darmstadt Germany

**Keywords:** cell culture medium, iron, low impurity, raw material impurity, recombinant protein product quality

## Abstract

Cell culture medium (CCM) composition affects cell growth and critical quality attributes (CQAs) of monoclonal antibodies (mAbs) and recombinant proteins. One essential compound needed within the medium is iron because of its central role in many cellular processes. However, iron is also participating in Fenton chemistry leading to the formation of reactive oxygen species (ROS) causing cellular damage. Therefore, this study sought to investigate the impact of iron in CCM on Chinese hamster ovary (CHO) cell line performance, and CQAs of different recombinant proteins. Addition of either ferric ammonium citrate (FAC) or ferric citrate (FC) into CCM revealed major differences within cell line performance and glycosylation pattern, whereby ammonium was not involved in the observed differences. Inductively coupled plasma mass spectrometry (ICP‐MS) analysis identified varying levels of impurities present within these iron sources, and manganese impurity rather than iron was proven to be the root cause for increased cell growth, titer, and prolonged viability, as well as altered glycosylation levels. Contrary effects on cell performance and protein glycosylation were observed for manganese and iron. The use of low impurity iron raw material is therefore crucial to control the effect of iron and manganese independently and to support and guarantee consistent and reproducible cell culture processes.

**Abbreviations**
CCM: cell culture medium
CGE‐LIF: capillary gel electrophoresis with laser‐induced fluorescence
CHO: Chinese hamster ovary
CQAs: critical quality attributes
DMT1: divalent metal‐iron transporter 1
DNA: deoxyribonucleic acid
EDTA: ethylenediaminetetraacetic acid
FAC: ferric ammonium citrate
FC: ferric citrate
Fe: iron
FeSOD2: iron bound to superoxide dismutase 2
GlcNAc: N‐acetylglucosamine
H_2_O: water
H_2_O_2_
: hydrogen peroxide
HNO_3_
: nitric acid
(HR)‐ICP‐MS: (high resolution) inductively coupled plasma mass spectrometry
HMW: high molecular weight
IFN‐γ: interferon gamma
IgG: immunoglobulin G
mAb: monoclonal antibody
Mn: manganese
NTBI: non‐transferrin‐bound iron
ROS: reactive oxygen species
SD: standard deviation
SEC‐UV: size exclusion chromatography coupled to an UV detector
SOD2: superoxide dismutase 2
TBI: transferrin‐bound iron
UPLC(‐MS): ultra‐performance liquid chromatography (coupled to a mass spectrometer)
VCD: viable cell density

## 1 INTRODUCTION

Chinese hamster ovary (CHO) cell culture processes are widely used for the production of recombinant proteins applied for the treatment of e.g. oncological and hematological diseases.^1^ Maintaining cell performance and quality of the final product is a common major goal and can be affected by cell line, process conditions, and cell culture medium (CCM) composition.^2^, ^3^ Chemically defined media with up to 100 components have been developed over the last decades, reducing lot‐to‐lot variations and complexity of the medium. Main components within these CCM are amino acids, carbohydrates, vitamins, lipids, inorganic salts, and trace elements.^4^, ^5^, ^6^ Each of these components can have a tremendous effect on cellular performance and critical quality attributes (CQAs) of the final protein and a good understanding of each of these component's role in cellular metabolism and protein expression is required.^6^ For instance, amino acids have been shown to increase final titer and modulate glycosylation profile of the produced protein when optimized in CCM,^7^, ^8^, ^9^ and several trace elements have been reported to impact CQAs of the final product, such as glycosylation, charge, and aggregation.^2^

Iron is an essential element for cellular processes. Many enzymes involved in energy metabolism, deoxyribonucleic acid (DNA) biosynthesis, and repair, or antioxidant functions use iron as a cofactor.^10^ Iron uptake in cell culture is regulated by two main mechanisms, the transferrin‐bound iron (TBI) uptake and the non‐transferrin‐bound iron (NTBI) uptake, depending on the availability of transferrin. In the presence of transferrin, two atoms of ferric iron are bound by it to form an iron‐transferrin complex. This complex is then recognized by transferrin receptor 1 and a receptor‐mediated endocytosis of TBI follows. Within the endosome, ferric iron is released from transferrin due to acidification and the iron is subsequently reduced to ferrous iron by an endosomal ferrireductase. In the NTBI uptake, several surface ferrireductases are involved, reducing NTBI to its ferrous form before iron is imported to the cell by transporters such as the divalent metal‐ion transporter 1 (DMT1).^11^, ^12^

The capability of iron to take part in redox reactions makes it a crucial transition metal for cellular functions. However, iron can catalyze Fenton reactions resulting in the formation of reactive oxygen species (ROS) that may lead to DNA, protein, or membrane damage.^11^, ^13^ The formation of ROS due to the presence of iron, which is commonly added in form of iron complexes such as ferric ammonium citrate (FAC), ferric citrate (FC), or ferric ethylenediaminetetraacetic acid (EDTA), can increase the degradation rate of media components in CCM.^14^, ^15^, ^16^

Altering the iron concentration within CCM can have different effects on cell performance and final CQAs of the product. Increasing iron concentrations were shown to improve overall cell growth and productivity of a CHO cell culture.^15^, ^17^ However, an increase in CCM iron concentration was also demonstrated to correlate with increased color formation of the recombinant proteins produced in CHO cells, which also correlated with an increased level of acidic charge variants. Both observations are likely to be related to oxidation effects within the recombinant protein caused by iron‐generated ROS.^14^, ^18^ ROS production due to iron was also identified to cause oxidative stress enhancing protein aggregation, for example in neuronal or lens crystallin proteins.^19^, ^20^ In other studies, the effect of iron on glycosylation macroheterogeneity was investigated. The addition of iron to CCM was reported to increase site‐occupancy of the glycoprotein tissue plasminogen activator.^21^ Moreover, a constant glycosylation pattern was detected for interferon gamma (IFN‐γ) upon iron citrate addition to the medium compared to the non‐supplemented version, in which increased levels of non‐glycosylated IFN‐γ were observed.^22^ Furthermore, the addition of iron to CCM was shown to significantly increase galactosylation of the recombinant glycoprotein, which has been disclosed in US Patent No. 9598667B2.^23^

In this study, the impact of iron in CCM on cell performance and product quality (aggregation and glycosylation profile) was investigated. Fed‐batch data for a CHO K1 cell line revealed major differences in cell performance and glycosylation profile of the recombinant monoclonal antibody (mAb) upon usage of different iron sources. Inductively coupled plasma mass spectrometry (ICP‐MS) characterization of the sources was performed to determine elemental impurity levels within the raw material. Among all impurities, manganese was identified as the root cause for improved cell performance and altered glycosylation level of the recombinantly produced protein. The effect of manganese‐contaminated iron source was further studied for another CHO K1 cell line as well as for a CHOZN® clone. Altogether, the results demonstrate the importance and need of low impurity iron sources in CCM to ensure consistent and reproducible cell culture processes.

## 2 MATERIAL AND METHODS

### 2.1 Reagents and cell lines

Ferric ammonium citrate (FAC), ferric citrate (FC_Purch_) and manganese (II) chloride were purchased from Merck, Darmstadt, Germany. Ammonium chloride was purchased from Sigma Aldrich, St. Louis, MO. Ferric citrate low impurity (FC_Synt_) was synthesized in‐house.

Two CHO K1 cell lines (1 and 2) producing two different recombinant immunoglobulins G (IgGs) (mAb1 and mAb2, respectively) and one CHOZN® clone (cell line 3) producing a fusion protein were used within this study. Chemically defined Cellvento® 4CHO and 4Feed fed‐batch platform (Merck, Darmstadt, Germany) were used in this work.

### 2.2 Cell culture experiments

Fed‐batch experiments were performed in 50 ml spin tubes (TPP, Trasadingen, Switzerland) with vented cap at 37°C, 5% CO_2_, 80% humidity and with an agitation speed of 320 rpm. Iron deficient Cellvento® 4CHO medium was used, whereas different amounts of iron in the form of either FAC or FC were added. Seeding density was 2 × 10^5^ cells/ml in a working volume of 30 ml. Cellvento® 4Feed was added on day 3, 5, 7, 10, 12, and 14 with a feeding strategy of 1.5, 3, 3, 3, 3, and 3% (v/v), respectively (cell line 1 and 3). For cell line 2, 3% (v/v) were added on day 3, 5, 10, 12, and 14 and 6% (v/v) on day 7. Glucose (400 g/L) was fed on demand to up to 6 g/L during the week and up to 13 g/L before the weekend. Viable cell density (VCD) and viability were measured with the Vi‐CELL™ XR 2.04 (Beckman Coulter, Fullerton, CA). Glucose, titer, iron and ammonium concentrations were analyzed with the Cedex Bio HT (Roche, Mannheim, Germany) after centrifugation of the sample for 5 min at 4500 rpm (2287 g).

### 2.3 Antibody purification and CQAs analysis

Antibodies and fusion proteins were purified from the cell culture supernatant by using protein A PhyTips® (PhyNexus Inc., San Jose, CA). Aggregation profile was analyzed using size exclusion chromatography coupled to an UV detector (SEC‐UV) using an Acquity ultra‐performance liquid chromatography (UPLC) (Waters, Milford, MA) and a TSK gel SuperSW series column (Tosoh Bioscience, Griesheim, Germany) at room temperature. 10 μl of sample, adjusted to 1 mg/ml with storage buffer (85% (v/v) of 30 mM citric acid pH 3.0 and 15% (v/v) of 0.375 M Tris Base pH 9.0), were applied to the system at a flow rate of 0.35 ml/min. 0.05 M sodium phosphate, 0.4 M sodium perchlorate solution adjusted to pH 6.3 was used as mobile phase. Glycosylation analysis was performed either by capillary gel electrophoresis with laser‐induced fluorescence (CGE‐LIF) for the two antibodies or by UPLC coupled to a mass spectrometer (UPLC‐MS) for the fusion protein as described elsewhere.^24^

### 2.4 Iron source characterization

The detection and quantification of trace elements within iron sources was performed using ICP‐MS. Sample preparation was carried out by microwave‐assisted digestion involving nitric acid. Indium was used as internal standard throughout all analyses. Either a semiquantitative elemental screening method was performed involving the adaption of the system response curve as intensity per ng/ml of analyte by a single point calibration using an ELAN 6000 (PerkinElmer Inc., Waltham, MA), or a quantification by external calibration using an ELEMENT 2™ high resolution (HR)‐ICP‐MS (ThermoFisher, Waltham, MA) was done. In both cases, the recovery rate was determined by spiking the samples with a known amount of analyte.^25^, ^26^

### 2.5 Statistical analysis

Data are expressed as means ± standard deviation (SD) of six biological replicates unless stated otherwise. Graphic analysis was performed with GraphPad Prism 8 Software (GraphPad Software Inc., San Diego, CA). Statistical analysis was performed by the non‐parametric Kruskal‐Wallis test for multiple‐group comparison with subsequent Dunn's test. *p*‐values smaller than 0.01 or 0.001 were considered significant.

## 3 RESULTS

### 3.1 Effect of increasing iron amounts in CCM on cell performance and IgG quality attributes

**FIGURE 1:**
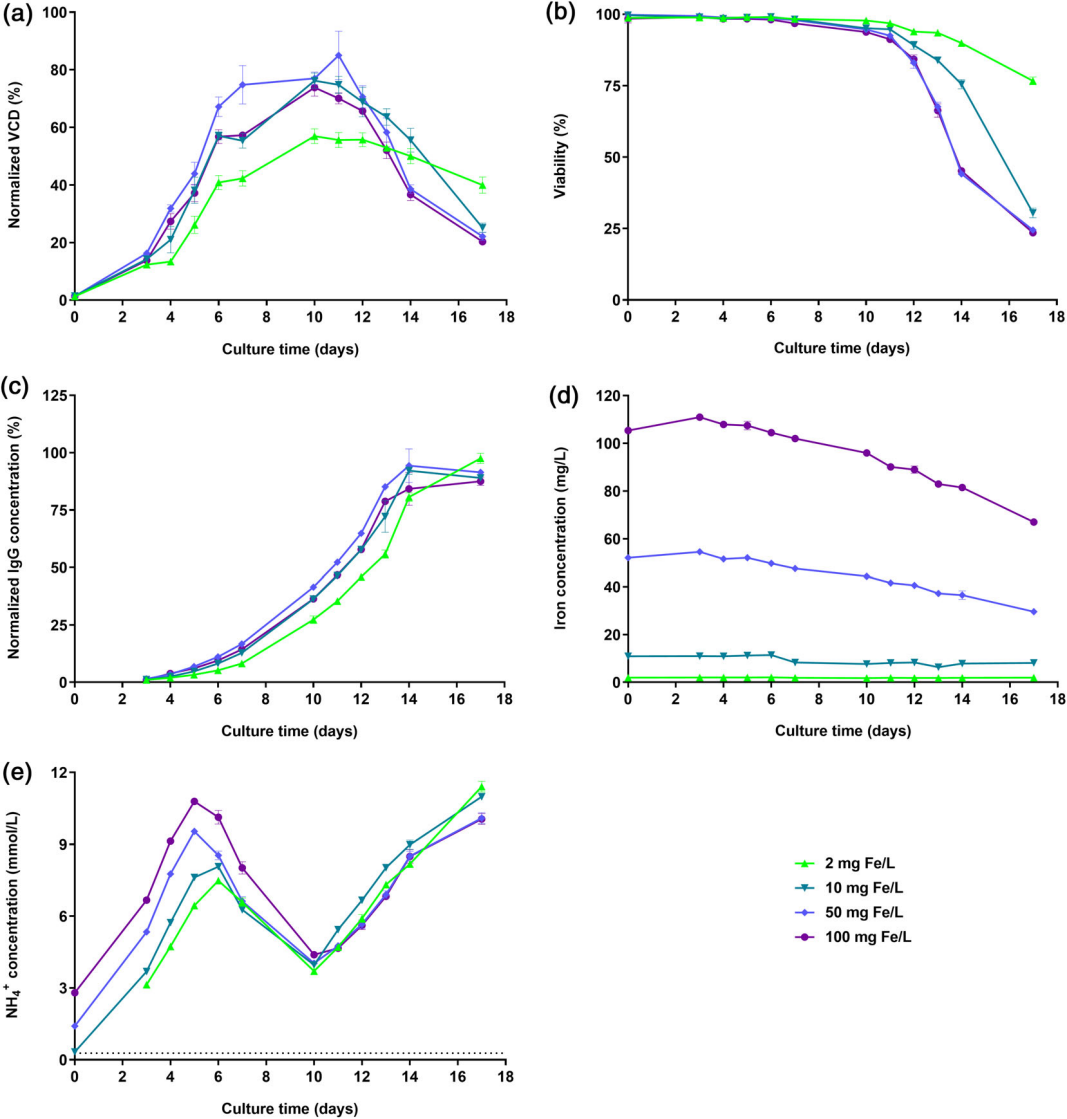
Effect of FAC iron source dose response in CCM on cell performance of cell line 1 in fed‐batch process. CHO K1 cells were cultivated in medium supplemented with either 2, 10, 50, or 100 mg Fe/L (FAC). (a) VCD in % normalized to the highest value. (b) Viability in %. (c) IgG concentration in % normalized to the highest value. (d) Iron concentration in mg/L. (e) Ammonium concentration in mmol/L. The horizontal dotted line in panel (e) represents the lower limit of detection. Data present mean ± SD of six biological replicates

To understand whether increasing amounts of iron in CCM impact cell performance and the quality of the produced recombinant antibody, a small‐scale iron dose response fed‐batch experiment was performed in spin tubes with cell line 1. Therefore, increasing amounts of FAC, a commonly used iron source in CCM, were spiked to iron deficient Cellvento® 4CHO to obtain iron concentrations of 2, 10, 50, and 100 mg/L. As shown in Figure 1, the cell performance was significantly impacted by the addition of increasing FAC concentrations. For instance, VCD on day 10 was higher for 10, 50, and 100 mg Fe/L compared to 2 mg Fe/L (absolute increase of 19.2%, 20.0%, and 16.8%, respectively) (Figure 1a). However, the increase in cell growth led to a faster decrease in viability (Figure 1b). The highest final titer (D17) was detected for 2 mg Fe/L even though the IgG concentration was higher for 50 mg Fe/L until D14 (Figure 1c), suggesting that 50 mg Fe/L may be more suitable for commercial processes where the harvest is commonly performed at viabilities above 80%. The iron concentration decreased significantly during the course of the fed‐batch process for 10, 50, and 100 mg Fe/L, and the absolute decrease in iron concentration over time increased with increasing iron concentration (Figure 1d). Since FAC was used as iron source, higher ammonium concentrations were detected at day 0 for higher applied iron concentrations in the medium. However, the produced metabolic ammonium amount for all tested conditions was similar for the following days (Figure 1e), indicating that the starting concentration is not likely to impact cell metabolism. Iron deficient medium led to no cell growth and no titer production highlighting the necessity of iron in CCM to maintain essential cellular functions (data not shown).

**FIGURE 2:**
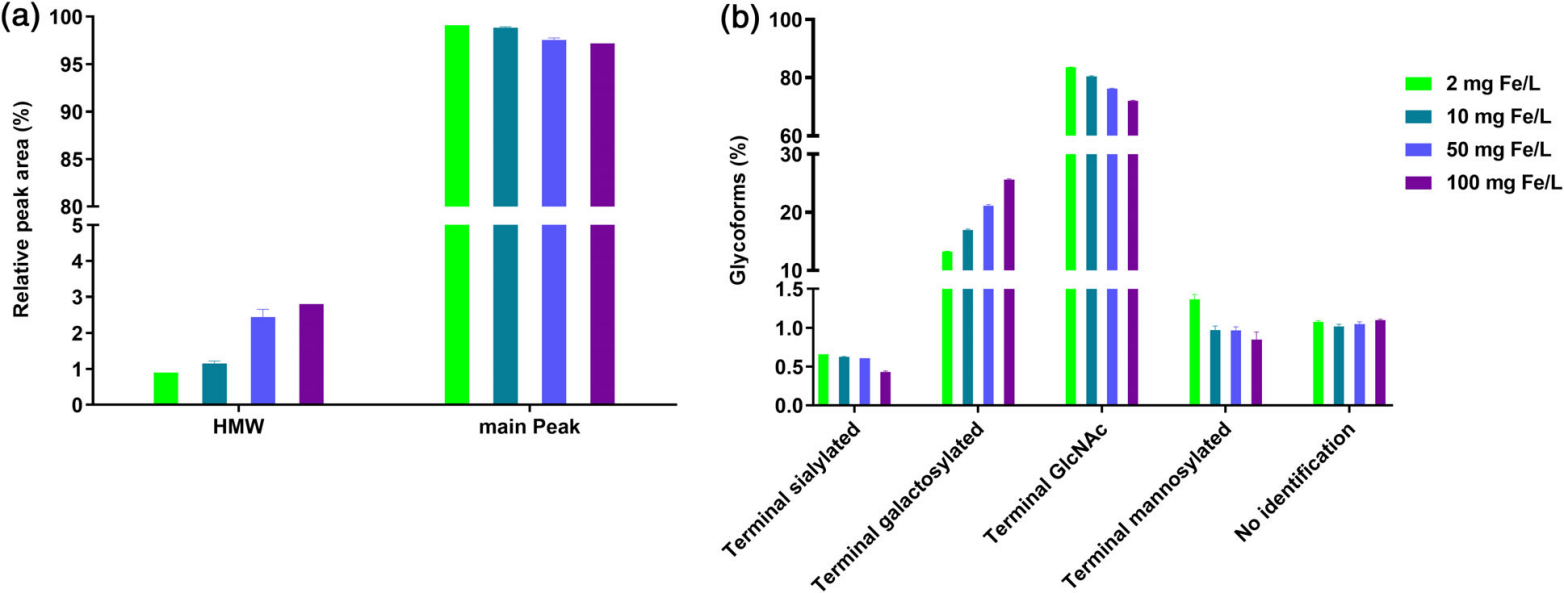
CQAs of mAb1 produced by cell line 1 on day 10 of FAC dose response fed‐batch process. (a) HMW and main peak level in %. (b) N‐glycosylation forms (terminal sialylated, terminal galactosylated, terminal GlcNAc, and terminal mannosylated) in %. Data are mean ± SD of two replicate pools (each with three biological replicates)

To investigate the effect of increasing FAC concentrations on mAb1 CQAs, aggregation and glycosylation profiles were determined by SEC‐UV and CGE‐LIF, respectively. Therefore, samples of day 10 of the fed‐batch process were analyzed for which cell culture viability was comparable and still very high for all conditions allowing the evaluation of only iron source‐related effects on CQAs. For the aggregation profile, a small dose‐dependent increase in high molecular weight (HMW) species from 0.9% (2 mg Fe/L) to 2.8% (100 mg Fe/L) was observed with increasing FAC amounts in the medium, which inversely correlated with the detected decrease of the main peak (Figure 2a). Glycosylation pattern demonstrated a significant dose‐dependent effect. With increasing iron amounts in the medium from 2 to 100 mg/L, the level of terminal galactosylated species increased from 13.3 to 25.6%, which inversely correlated with the observed decrease in terminal N‐acetylglucosamine (GlcNAc) level. Detected terminal sialylation, mannosylation and non‐identifiable species within mAb1 were below 0.7%, 1.4%, and 1.2%, respectively (Figure 2b) and were not impacted by iron. Altogether, the results indicate that increasing FAC concentrations are responsible for an increase in maximal VCD and a decrease in cell culture viability, while causing a slight increase in HMW species and an elevated level of terminal galactosylation species for mAb1.

### 3.2 Effect of ammonium on cell performance and IgG quality attributes

**FIGURE 3:**
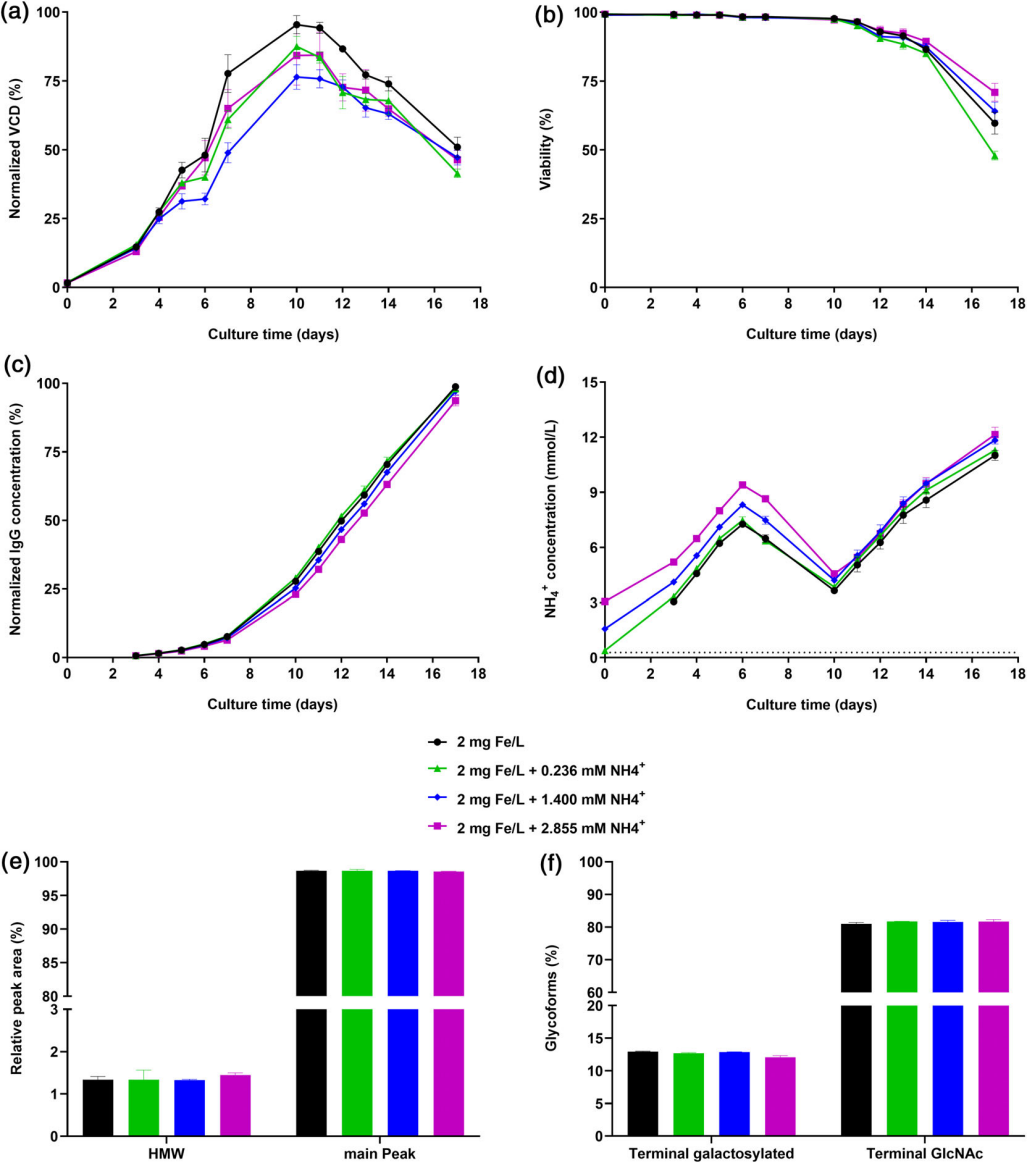
Effect of ammonium dose response in CCM on cell performance of cell line 1 and on CQAs of mAb1. CHO K1 cells were cultivated in medium supplemented with 2 mg Fe/L (FAC) and supplemented with either 0 (=positive control), 0.236, 1.400, or 2.855 mM ammonium in the form of ammonium chloride. CQAs of mAb1 were determined on day 10 of fed‐batch process. (a) VCD in % normalized to the highest value. (b) Viability in %. (c) IgG concentration in % normalized to the highest value. (d) Ammonium concentration in mmol/L. (e) HMW and main peak level of mAb1 in %. (f) N‐glycosylation forms (terminal galactosylated and terminal GlcNAc) of mAb1 in %. The horizontal dotted line in panel (d) represents the lower limit of detection. Data present mean ± SD of four (a, b, c, and d) or two (e and f) biological replicates

To investigate whether ammonium, introduced by FAC, is responsible for the observed changes in cell performance and CQAs, 0, 0.236 mM, 1.400 mM, and 2.855 mM of ammonium chloride were added to Cellvento® 4CHO medium containing 2 mg Fe/L (added in the form of FAC). This resulted in the same ammonium concentrations like for iron concentrations of 2, 10, 50, and 100 mg/L in form of FAC. Upon increasing amounts of supplemented ammonium, maximal VCD was decreased absolutely by 7.9%, 19.0%, and 11.1% in comparison to 2 mg Fe/L, but no dose‐dependent effect was observed (Figure 3a). No difference in viability was detected until D14 of the fed‐batch process (Figure 3b), whereas IgG concentration was only slightly decreased upon 2.855 mM ammonium compared to the other tested conditions (Figure 3c). Differences in ammonium concentration were observed for day 0, but the produced amount of metabolic ammonium was the same for all tested concentrations during the course of the fed‐batch (Figure 3d), indicating no significant impact of ammonium on cell metabolism. For the two tested CQAs aggregation and glycosylation (Figure 3e,f), no change was detected upon ammonium addition to CCM. Absolute differences for HMW and main peak species were below 0.2% and for glycosylation species below 0.9% for the tested conditions. Overall, these data suggest that ammonium present in FAC is neither responsible for increased cell performance, nor does it lead to elevated levels of HMW or terminal galactosylation species.

### 3.3 Impact of different iron sources, FAC and FC, on cell performance and IgG quality attributes

**FIGURE 4:**
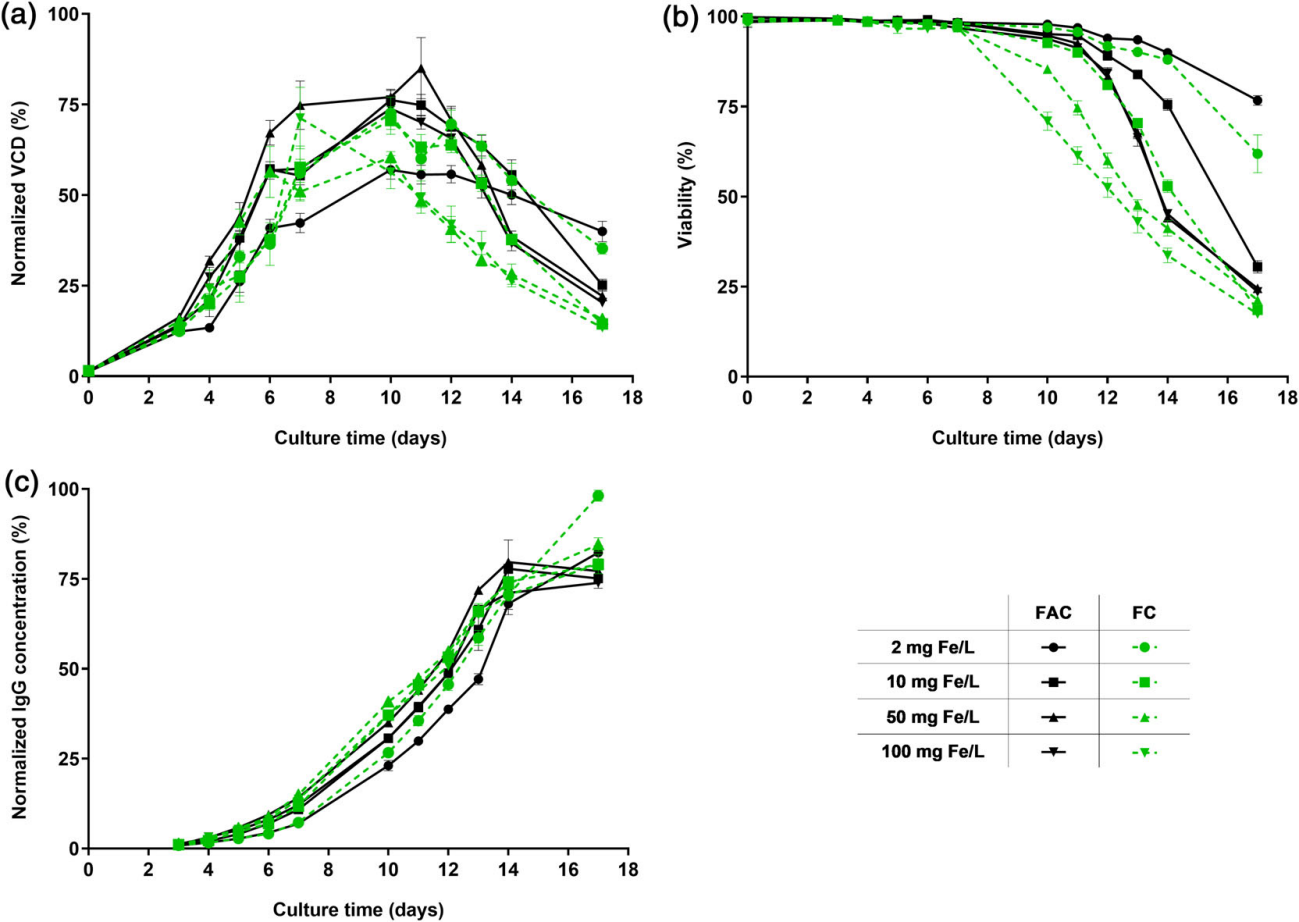
Effect of different iron sources, FAC and FC, in CCM on cell performance of cell line 1 in fed‐batch process. CHO K1 cells were cultivated in medium supplemented with either 2, 10, 50, or 100 mg Fe/L (FAC or FC). (a) VCD in % normalized to the highest value. (b) Viability in %. (c) IgG concentration in % normalized to the highest value. Data present mean ± SD of six biological replicates

Since increasing amounts of ammonium did not account for the observed differences in cell performance and CQAs upon increasing FAC concentrations, a second iron source, namely FC, was investigated. FC was selected due to the similar chelation strength and the absence of ammonium. Chosen iron concentrations were 2, 10, 50, and 100 mg/L and were added to iron deficient Cellvento® 4CHO medium. As shown in Figure 4, usage of FC as iron source in CCM caused a lower cell growth with a reduced maximal VCD of 7.4%, 28.9%, and 3.5% for 10, 50, and 100 mg Fe/L, respectively, compared to the corresponding FAC condition (Figure 4a). For both iron sources, a faster decrease in viability was detected with increasing iron concentrations, whereas an even faster decline in viability was observed for each tested FC concentration compared to the corresponding FAC condition (Figure 4b). Titer profiles showed only slight variations between the different tested iron sources (Figure 4c). Overall, the faster decline in VCD and viability upon usage of FC compared to FAC is independent of the iron level, suggesting that both iron sources present other differences.

To investigate whether FC has a different impact on mAb1 CQAs than FAC, glycosylation and aggregation profiles on day 10 of the fed‐batch process were analyzed. These samples were used to directly compare them to the previously analyzed samples upon FAC usage, although viability was already significantly different for FC on day 10. Table 1 summarizes the mAb1 glycosylation pattern of the two main species found upon usage of either FAC or FC in CCM. Whereas increasing levels of FAC in the CCM led to increased levels of terminal galactosylation with 13.3% for 2 mg Fe/L and up to 25.6% for 100 mg Fe/L, FC caused a decrease in terminal galactosylation species from 11.5% for 2 mg Fe/L to 8.0% for 50 mg Fe/L, which was inversely correlated with terminal GlcNAc levels. These data suggest again that the observed differences in glycosylation between FAC and FC are independent of the iron concentration and impacted by unidentified attributes between both iron sources.

**TABLE 1:** IgG glycosylation profile of mAb1 produced by cell line 1 on day 10 of iron dose response fed‐batch process upon usage of different iron sources, FAC and FC, in CCM. N‐glycosylation forms (terminal galactosylation and terminal GlcNAc) in %. Data are mean (±SD) of two replicate pools (each with three biological replicates)

Iron concentration in CCM in mg/L	Terminal galactosylation (%)		Terminal GlcNAc (%)	
	FAC	FC	FAC	FC
2	13.3 (±0.03)	11.5 (±0.11)	83.6 (±0.08)	84.6 (±0.01)
10	17.0 (±0.18)	9.9 (±0.08)	80.4 (±0.21)	86.4 (±0.01)
50	21.1 (±0.20)	8.0 (±0.04)	76.2 (±0.13)	87.7 (±0.05)
100	25.6 (±0.15)	8.6 (±0.04)	72.0 (±0.23)	86.7 (±0.01)

Aggregation profile upon FC usage showed the same small dose‐dependent increase in HMWs for increasing iron amounts like FAC (data not shown).

Altogether, the results obtained with increasing amounts of FAC and FC indicate that attributes other than iron and ammonium levels impact growth, viability, and IgG glycosylation.

### 3.4 Analysis of trace element impurities in FAC and FC

Since it is known from literature that iron sources can be contaminated with trace elements,^27^ the next step focused on trace element characterization of both iron sources because trace elements in general are known to influence cell performance and product quality.^27^, ^28^, ^29^, ^30^ Trace element impurity levels of FAC as well as FC were determined by ICP‐MS analyses. Table 2 summarizes all elemental impurities showing values above 3 μg/g for at least one iron source. Whereas some elements such as magnesium (Mg), aluminum (Al), calcium (Ca), titanium (Ti), vanadium (V), cobalt (Co), copper (Cu), zinc (Zn), and gallium (Ga) were only detected within FAC, other elements such as boron (B) and potassium (K) showed higher levels in FC and were not detected in FAC. For chromium (Cr), manganese (Mn), and nickel (Ni), higher levels were measured in FAC compared to FC. Since manganese is well described as a modulator of antibody galactosylation^30^ and the manganese amount present as impurity in the FAC iron source contributed to more than 94% of the total manganese concentration in the CCM formulation, this element was considered as highly relevant. Other impurities such as copper and zinc, that are also known to impact cell culture performance and CQAs,^28^, ^29^ contributed to less than 15% of the final medium concentration and were thus deemed less impactful and were not studied further. Based on these assessments, the increased manganese impurity in FAC raw material compared to FC iron source was hypothesized to be the major trigger for increased galactosylation and prolonged viability upon usage of FAC.

**TABLE 2:** Impurity profile of iron sources FAC and FC. The impurity characterization was performed by ICP‐MS, whereby the quantification was carried out by either a semiquantitative elemental screening method using a quadrupole‐based ICP‐MS or HR‐ICP‐MS utilizing an external calibration. Only elements showing values above 3 μg/g for at least one iron source are presented. B: boron, Mg: magnesium, Al: aluminum, K: potassium, Ca: calcium, Ti: titanium, V: vanadium, Cr: chromium, Mn: manganese, Co: cobalt, Ni: nickel, Cu: copper, Zn: zinc, Ga: gallium. All obtained calibration curves yielded a correlation coefficient of at least >0.999. * Values gained with HR‐ICP‐MS

μg/g	B	Mg	Al	*K	*Ca	*Ti	*V	*Cr	Mn	Co	*Ni	Cu	Zn	Ga
FAC	<1.0	150	110	<10	200	120	140	8.5	38	21	40	4	190	10
FC	5.6	<1.0	<5.0	17	<5.0	<5.0	<1.0	4.2	0.36	<1.0	0.8	<0.5	<25	<0.5

### 3.5 Effect of high and low manganese‐contaminated iron sources on cell performance and IgG quality attributes

**FIGURE 5:**
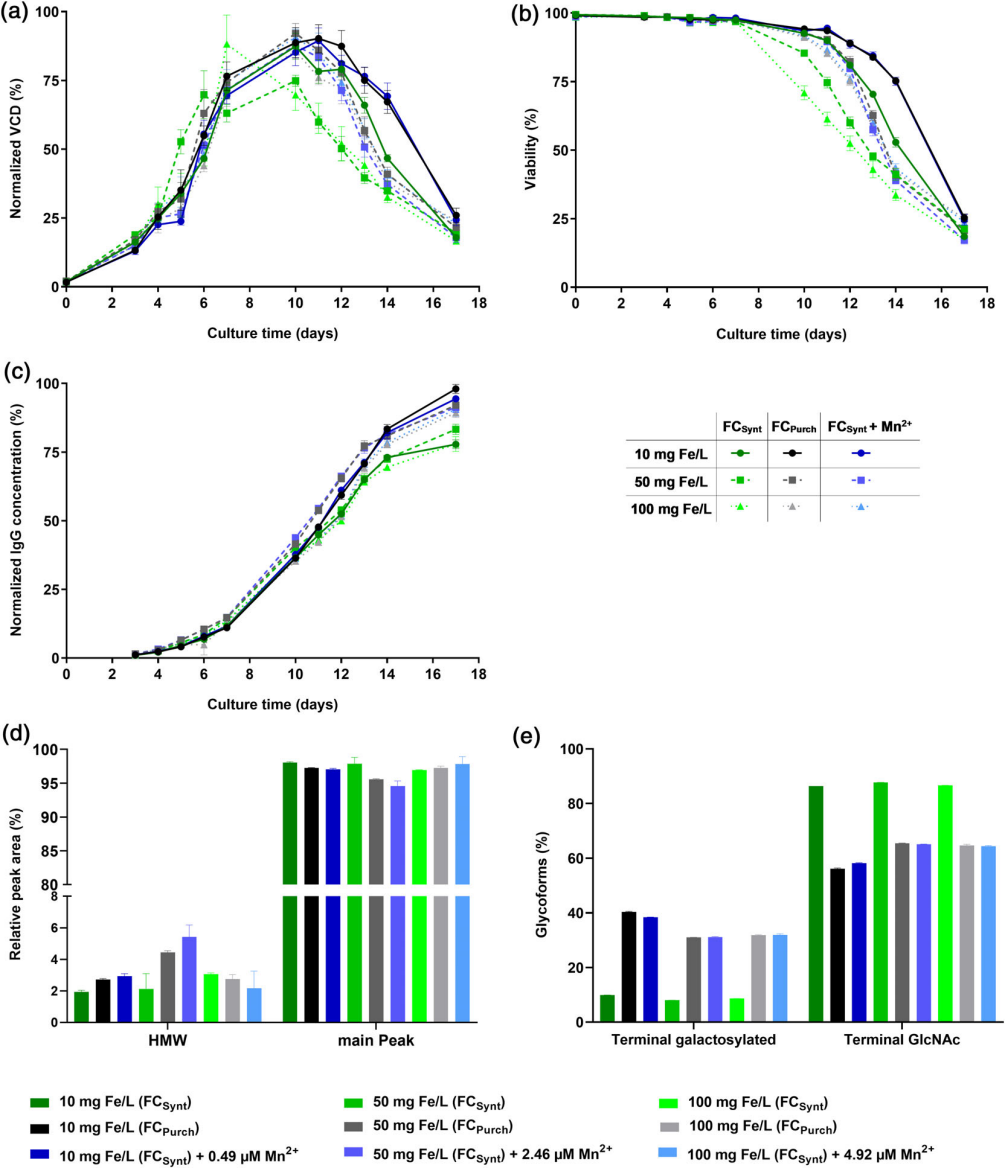
Effect of high and low manganese‐contaminated FC iron sources in CCM on cell performance of cell line 1 and on aggregation and glycosylation profile of mAb1. CHO K1 cells were cultivated in medium supplemented with either 10, 50, or 100 mg Fe/L (FC_Synt_ (low manganese impurity) or FC_Purch_ (high manganese impurity)). Additionally, for each tested iron concentration a third condition was prepared, where manganese was added to FC_Synt_ to achieve the same manganese concentration as present in FC_Purch_. CQAs of mAb1 were determined on day 10 of fed‐batch process. (a) VCD in % normalized to the highest value. (b) Viability in %. (c) IgG concentration in % normalized to the highest value. (d) HMW and main peak level of mAb1 in %. (e) N‐glycosylation forms (terminal galactosylated and terminal GlcNAc) of mAb1 in %. Data present mean ± SD of six biological replicates (a, b, and c) or two replicate pools (each with three biological replicates) (d and e)

Since ICP‐MS results showed a variation of manganese impurity within the used iron sources FAC and FC, a comparison of two similar iron sources, differing significantly in manganese impurity level, was studied further. As no low impurity FAC iron source was available on the market, all further studies were performed with FC, for which a low and high impurity iron source existed. These two FC iron sources, named in the following as FC_Purch_ (high level of manganese impurity; 530 μg Mn/g FC [impurity profile presented in Table S1]) and FC_Synt_ (low level of manganese impurity; 0.36 μg Mn/g FC), were supplemented to iron deficient CCM in three different iron concentrations (10, 50, and 100 mg Fe/L) and a fed‐batch with cell line 1 was performed. Additionally, for each chosen iron concentration, a third condition was tested, where manganese was supplemented to FC_Synt_ (FC_Synt_ + Mn^2+^) to match the concentration present in the corresponding FC_Purch_ condition. Results (Figure 5) indicate a higher cell growth for FC_Purch_ compared to the respective FC_Synt_ independently of the iron concentration, whereas the supplementation of manganese to FC_Synt_ led to a recovery of the VCD similar to the manganese impure FC_Purch_ (Figure 5a). Upon usage of either FC_Purch_ or FC_Synt_ supplemented with manganese, a prolonged viability was observed in comparison to the respective FC_Synt_ condition (Figure 5b). IgG concentrations for FC_Purch_ and FC_Synt_ supplemented with manganese were higher compared to IgG concentrations detected for FC_Synt_ indicating the positive impact of manganese on productivity (Figure 5c). mAb1 HMW species level was lower than 5.5% for all tested conditions and the absolute difference was smaller than 3.3% for each tested iron concentration. The detected main peak level for mAb1 was above 94.5% for all tested conditions (Figure 5d). Glycosylation results indicate that manganese, either present as impurity or supplemented to CCM, significantly increased terminal galactosylation levels. Average absolute increase in terminal galactosylation species for FC_Purch_ and FC_Synt_ + Mn^2+^ in comparison to FC_Synt_ was above 22.5%. For FC_Synt_, a slight decrease in terminal galactosylated level with increasing iron concentration was observed (Figure 5e).

**FIGURE 6:**
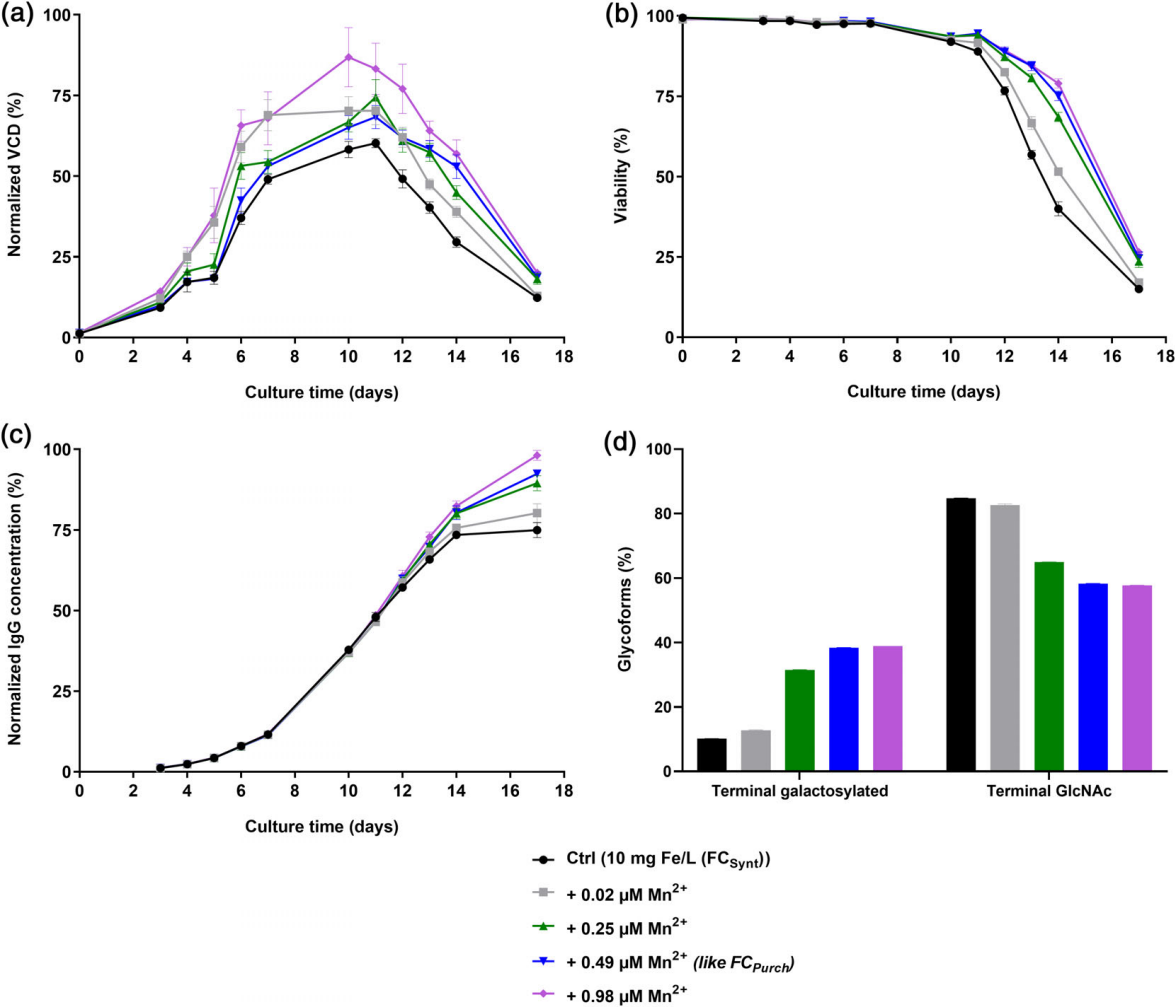
Effect of manganese dose response in CCM on cell performance of cell line 1 and on glycosylation profile of mAb1. CHO K1 cells were cultivated in medium supplemented with 10 mg Fe/L (FC_Synt_ (low manganese impurity)) and 0 (=positive control), 0.02, 0.25, 0.49, or 0.98 μM Mn^2+^ in the form of manganese (II) chloride. N‐glycosylation profile of mAb1 was determined on day 10 of fed‐batch process. (a) VCD in % normalized to the highest value. (b) Viability in %. (c) IgG concentration in % normalized to the highest value. (d) N‐glycosylation forms (terminal galactosylated and terminal GlcNAc) of mAb1 in %. Data are mean ± SD of six biological replicates (a, b, and c) or two replicate pools (each with three biological replicates) (d)

In order to investigate the positive effect of manganese in more detail, increasing amounts of manganese were supplemented to FC_Synt_ containing CCM with 10 mg Fe/L and a small‐scale fed‐batch experiment with cell line 1 was performed. Increasing amounts of manganese in the medium led to an overall increased cell performance (Figure 6). Maximal VCD on day 10 for 0.98 μM Mn^2+^ was significantly increased (+49.1%, *p*‐value <0.001, non‐parametric Kruskal‐Wallis test for multiple‐group comparison with subsequent Dunn's test) compared to the FC_Synt_ control (Figure 6a). Furthermore, increasing amounts of manganese in CCM led to prolonged viabilities (Figure 6b). Significantly higher final titers compared to the non‐supplemented FC_Synt_ condition were detected for 0.49 μM Mn^2+^ (+23.3%, *p*‐value <0.01) and 0.98 μM Mn^2+^ (+30.9%, *p*‐value <0.001), whereas titer profiles until D14 were comparable (Figure 6c). mAb1 glycosylation results indicate that increasing amounts of manganese led to a significant increase in terminal galactosylation species. Absolute difference for terminal galactosylation level for 0.02, 0.25, and 0.49 μM Mn^2+^ in comparison to the positive control FC_Synt_ were determined to be 2.6%, 21.4%, and 28.2%, respectively, whereas no further increase in terminal galactosylation was detected for 0.98 μM Mn^2+^ in comparison to 0.49 μM Mn^2+^ (Figure 6d). Absolute differences in mAb1 aggregation profile on day 10 for all tested conditions were determined to be less than 0.8% with an average level of HMWs of 3.0% (data not shown). These results demonstrate once more the positive effect that manganese can have on cell performance, but also shows the impact of manganese to significantly increase terminal galactosylation level and thus the necessity to keep manganese levels constant for the production of recombinant proteins.

### 3.6 Effect of high and low manganese‐contaminated FC iron sources on further cell lines

In order to investigate whether other cell lines show differences in cell performance or CQAs for different levels of manganese impurity in iron sources, further small‐scale fed‐batch experiments were performed with two other CHO cell lines. Therefore, iron deficient Cellvento® 4CHO was supplemented with either FC_Synt_ or FC_Purch_. Additionally, for each iron concentration, a third condition was tested, where manganese was added to FC_Synt_ to match the concentration present in FC_Purch_ containing CCM. For each cell line, two different iron concentrations within the medium were studied.

**FIGURE 7:**
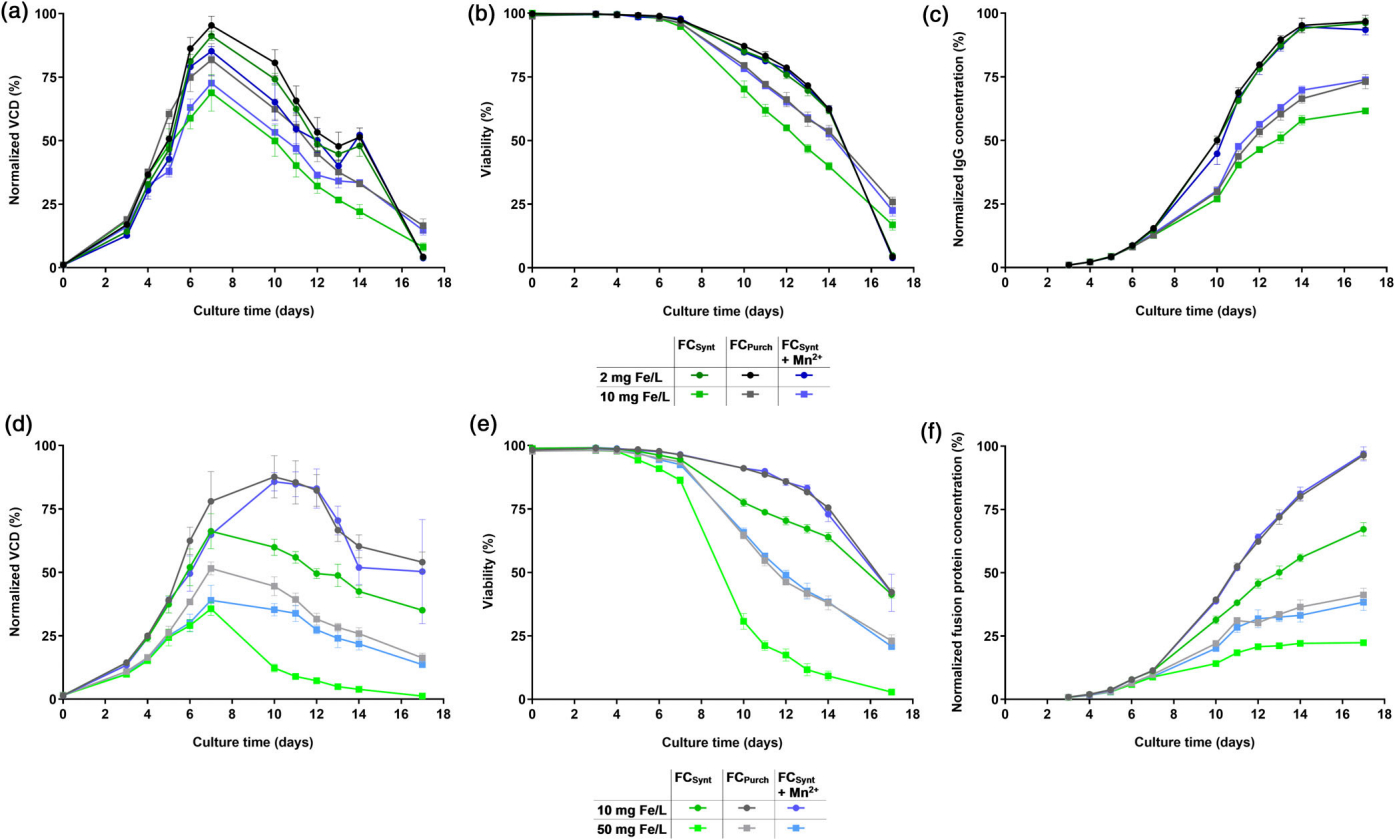
Effect of high and low manganese‐contaminated FC iron sources in CCM on cell performance of cell line 2 and 3 in fed‐batch process. CHO K1 cells (cell line 2) and CHOZN® cells (cell line 3) were cultivated in medium supplemented with either 2, 10, or 50 mg Fe/L (FC_Synt_ (low manganese impurity) or FC_Purch_ (high manganese impurity)). Additionally, for each tested iron concentration a third condition was prepared, for which manganese was added to FC_Synt_ to achieve the same manganese concentration as present in FC_Purch_. Cell line 2: (a) VCD in % normalized to the highest value. (b) Viability in %. (c) IgG concentration in % normalized to the highest value. Cell line 3: (d) VCD in % normalized to the highest value. (e) Viability in %. (f) Fusion protein concentration in % normalized to the highest value. Data present mean ± SD of four biological replicates

For cell line 2, a higher maximal VCD was obtained for 10 mg Fe/L FC_Purch_ and FC_Synt_ plus manganese supplementation compared to 10 mg Fe/L FC_Synt_, whereas for 2 mg Fe/L, only minor changes in cell growth were observed (Figure 7a). Similarly, prolonged viability of cell culture was detected for 10 mg Fe/L FC_Purch_ and FC_Synt_ plus manganese addition in comparison to FC_Synt_, whereas no difference was detected for 2 mg Fe/L containing conditions (Figure 7b). A difference in titer between the high and low manganese containing conditions was only detected for 10 mg Fe/L, with more than 18% increased final titer in comparison to the low manganese impurity FC_Synt_ (Figure 7c).

For cell line 3, a higher cell growth was observed for FC_Purch_ and FC_Synt_ + Mn^2+^ compared to FC_Synt_ with an increase in maximal VCD of more than 32% and 9% for 10 and 50 mg Fe/L, respectively (Figure 7d), whereas a prolonged viability upon the presence of manganese was also observed for this cell line independently of the tested iron concentration (Figure 7e). Increased titers for the high manganese conditions in comparison to FC_Synt_ were observed for both tested iron concentrations (Figure 7f). Altogether, these results demonstrate that elevated levels of manganese in CCM led to an increased cell performance for both, cell line 2 and cell line 3.

**FIGURE 8:**
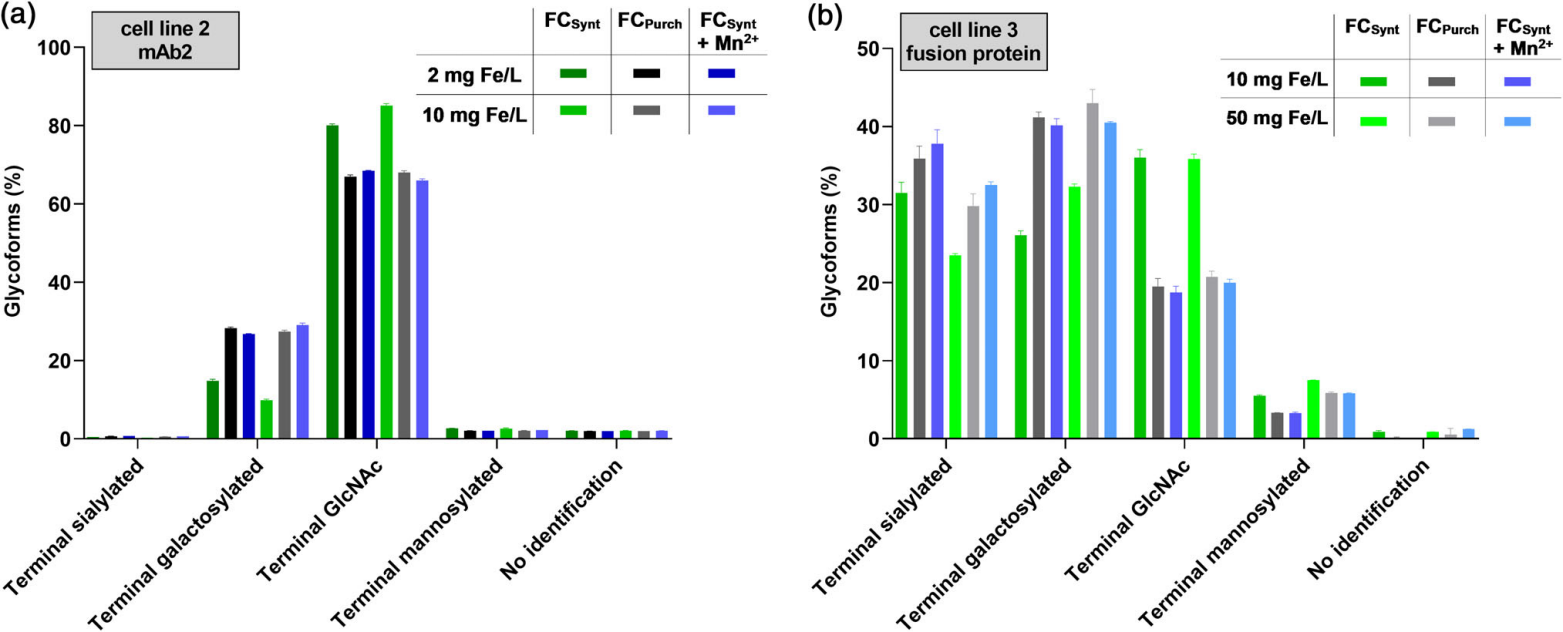
Glycosylation profile of mAb2 and fusion protein produced by cell line 2 and cell line 3, respectively, on day 10 of fed‐batch process upon usage of high and low manganese‐contaminated FC iron sources (FC_Purch_ and FC_Synt_) in CCM. (a) N‐glycosylation forms (terminal sialylated, terminal galactosylated, terminal GlcNAc, and terminal mannosylated) of mAb2 in %. (b) N‐glycosylation forms (terminal sialylated, terminal galactosylated, terminal GlcNAc, and terminal mannosylated) of fusion protein in %. Data are mean ± SD of two replicate pools (each with two biological replicates)

Study of the glycosylation and aggregation profile of mAb2 produced by cell line 2 and the fusion protein produced by cell line 3 was performed thereafter. Glycosylation analysis was performed by CGE‐LIF for mAb2 and UPLC‐MS for the fusion protein. For cell line 2 (Figure 8a), usage of FC_Purch_ and FC_Synt_ supplemented with manganese led to a significant increase in terminal galactosylated species of mAb2 in comparison to FC_Synt_ (absolute increase of more than 13%). Usage of FC_Synt_ alone led to a slightly decreased level of terminal galactosylation species for 10 mg Fe/L (9.8%) in comparison to 2 mg Fe/L (14.8%), similarly to the results observed for mAb1. Glycosylation profile of fusion protein produced by cell line 3 (Figure 8b) indicated that a high level of manganese present as contamination in FC_Purch_ increased terminal sialylated species and terminal galactosylated species significantly in comparison to FC_Synt_, whereas the glycosylation profile of FC_Purch_ was restored upon addition of manganese to FC_Synt_. Independently of the manganese contamination, an increase in FC_Synt_ concentration from 10 to 50 mg Fe/L led to an absolute decrease in terminal sialylation species of 8.0% and to an absolute increase in terminal galactosylation species of 6.2% suggesting an iron‐dependent effect, which is in terms of galactosylation opposite compared to mAb1 and mAb2. Furthermore, since an increase in manganese increased terminal sialylation level but an increase in iron concentration decreased terminal sialylation level, an opposite effect of both elements on sialylation is suggested. For all tested iron concentrations, neither iron, nor manganese demonstrated an impact on aggregation levels (data not shown).

Overall, these results show that manganese and iron have opposite effects on cell performance and protein glycosylation, whereas the effect on mAb2 and fusion protein galactosylation is contrary, demonstrating the need of adding iron and manganese independently of each other to CCM to decouple their effects in cell culture processes.

## 4 DISCUSSION

Iron is an essential trace element needed in CCM to maintain and regulate cellular functions. It plays a key role as a cofactor in many enzymatic processes due to its capability to take part in redox reactions. However, the redox cycling properties of iron may lead to the formation of ROS, which induce oxidative stress to the cells.^10^, ^13^ In this study, we therefore evaluated the impact of iron in CCM on cell performance and CQAs. FAC iron source dose response in CCM for cell line 1 in a fed‐batch process indicated significant changes in cell growth, viability, and glycosylation profile of mAb1. Those differences were proven to be independent of ammonium introduced by different amounts of FAC. Furthermore, comparison of FAC and FC iron source also demonstrated significant differences in cell performance and mAb1 glycosylation profile indicating that other factors than iron concentration were responsible for altered viability and glycosylation level. ICP‐MS analysis of iron raw material revealed differences in trace element impurity profiles. Thus, it was hypothesized that trace element contaminations within the iron sources were responsible for the varying effects between FAC and FC. Among all present impurities, manganese was identified as the root cause for the observed effects within this study.

Increasing amounts of manganese, introduced to CCM either by MnCl_2_ or in form of impurities, prolonged viability and increased titer significantly for all tested cell lines. Manganese is a known cofactor for mitochondrial superoxide dismutase 2 (SOD2), an enzyme involved in stress response.^27^, ^31^ By dismutating ROS created during mitochondrial respiration, SOD2 is involved in antioxidant defense and thereby protects cells from apoptosis.^32^ Furthermore, non‐proteinaceous complexes of manganese were demonstrated to perform antioxidant functions. For instance, manganese bound to small molecules such as phosphate or carboxylates was reported to remove toxic superoxide by forming first a MnO_2_^+^ complex, which is subsequently disproportionated into hydrogen peroxide (H_2_O_2_).^33^, ^34^, ^35^, ^36^ Thus, within this study, manganese might have increased the cellular antioxidant capacity of the cell and thereby improved performance.

An opposite effect on cell performance was observed for iron. Usage of increasing FC_Synt_ concentrations in CCM caused a decrease in cell growth and titer, as well as a faster drop in cell culture viability. This suggests that an iron‐mediated cell death might be a root cause for the loss of cell performance. For instance, several enzymes responsible for ROS formation such as cytochrome P450, xanthine oxidase, or enzymes involved in the mitochondrial electron transport chain, are using iron or iron–sulfur [Fe–S] clusters and thereby contributing to ROS‐mediated cell death.^37^ Furthermore, in eukaryotic organisms, apoptotic cell death was demonstrated to be mainly triggered by iron generated mitochondrial ROS leading to oxidative stress.^38^

Comparing the effects of manganese and iron on cell performance, the data suggest a possible opposite impact of both metals on cellular oxidative stress regulation affecting cell performance. Although both, manganese and iron, are metals involved in redox chemistry, both metals have different potentials to cause oxidative stress. Whereas iron is known to generate very oxidizing species through the Fenton reaction,^39^ manganese has a higher reduction potential than iron and is thus less reactive.^33^ Furthermore, SOD2 is commonly known as an antioxidant enzyme using manganese as a cofactor, however, recent publications suggest an iron‐dependent inactivation of antioxidant SOD2 activity.^33^, ^40^ For instance, incorporation of iron within mitochondrial SOD2 was reported to mediate mitochondrial dysfunction by lowering respiratory activity and increasing lipid peroxidation, thus decreasing the antioxidant defense mechanisms of the cells. Furthermore, reduced oxidative phosphorylation within the mitochondria and an increased flux through glycolysis as a result of a shift in cellular metabolism was observed.^10^, ^40^, ^41^ Iron bound to SOD2 (FeSOD2) revealed an alternative peroxidase activity for the enzyme, that was demonstrated by oxidizing the non‐fluorescent compound Amplex Red to the fluorescent resorufin in the presence of an excess of H_2_O_2_. FeSOD2 was thus suggested to mediate oxidation reactions, causing oxidative damage, and free radical formation within the cell.^40^, ^42^, ^43^ Therefore, iron and manganese within this study might have caused an opposite effect on the antioxidant defense capacity of SOD2 within the cell. Decoupling the effect of both trace elements in CCM by using low impurity iron source is thus important in order to determine optimal concentrations for both elements independently to achieve good cell performance.

Although previous reports have demonstrated that supplementation of copper to CCM increases cell growth and titer,^28^, ^44^ whereas zinc supplementation was demonstrated to decrease galactosylation level of the recombinant protein,^29^ none of those trace elements impacted the results observed within this study. Even though the impurity profile for both used iron sources FAC and FC_Synt_ showed significant variations for other trace elements besides manganese, a direct comparison between FAC and manganese supplemented FC_Synt_ proved that no trace elements other than manganese altered cell performance and CQAs upon usage of FAC or FC (Figure  S1).

Whereas manganese was demonstrated to increase terminal galactosylation and terminal sialylation of mAb1/mAb2 and fusion protein, respectively, which is a well characterized effect in literature,^7^, ^24^, ^30^, ^45^ iron revealed an opposite effect on glycosylation. Usage of increasing amounts of low impurity iron source FC_Synt_ led to a slight decrease of terminal galactosylation species for mAb1 and mAb2. An effect of iron on glycosylation was already described in literature, where the spiking of iron and copper salt solution to a bioreactor process decreased IgG3:κ terminal galactosylation level.^46^ The underlying mechanism has not been established but in general, metals are known to impact enzymes in the glycosylation pathway. For instance, ß‐1,4‐galactosyltransferase was shown to have two metal binding sites with high affinity for manganese^47^ and addition of metal ions such as zinc or cobalt resulted in the formation of an enzyme‐metal‐complex that reduced ß‐1,4‐galactosyltransferase activity.^48^ Thus, in this study, it is possible that iron also caused the formation of an inhibitory enzyme‐metal‐complex leading to a decrease in terminal galactosylation species.

Contrary to the observed effect of iron on mAb1 and mAb2 terminal galactosylation, usage of increasing amounts of low impurity FC_Synt_ had a different impact on the glycosylation profile of the fusion protein presenting significant levels of sialylation. Upon increasing FC_Synt_ concentrations, a significant decrease in terminal sialylated species and increase in terminal galactosylation level was detected. One hypothesis is that the sialylation level decreased with higher FC_Synt_ concentration due to a loss of cell viability. Such a correlation was already reported as a consequence of released sialidase enzymes into the extracellular space due to cell lysis.^49^ In another study, the sialylation level of a CHO Fc‐fusion protein was decreased with increased oxidative stress and higher glycolytic metabolism, which also correlated with decreased cell culture viability.^50^ Another hypothesis for the decreased terminal sialylation level is that iron might inhibit sialyltransferases, similarly to the effect proposed for galactosyltransferase. Since the inhibitory effect was predominantly observed for sialic acid, the inhibitory potential of iron seems to be higher for sialyltransferase compared to galactosyltransferase. However, the effect of iron on galactosyltransferase and sialyltransferase activity, and its binding affinity to both enzymes needs to be further investigated. Altogether, the glycosylation results presented within this study indicate that manganese and iron caused different effects on galactosylation and sialylation. Thus, both elements need to be decoupled from each other to be evaluated and controlled independently.

For decoupling both elements, the usage of low impurity iron sources within CCM is needed and requires a careful understanding and management of the used iron sources. Several root causes for raw material impurities have been described. For instance, manufacturing processes for iron salts may use undefined starting materials coming from other industries and thus do not fit for a biopharmaceutical application. Utilization of impure solvents as well as the usage of contaminated equipment or leaching packaging during the manufacturing process might as well lead to raw material impurities and lot‐to‐lot inconsistencies.^51^, ^52^

Since raw material variability is very well known to impact CCM performance,^27^, ^46^, ^53^ appropriate control systems for synthesized raw materials are essential to reduce the variability due to trace metals.^54^ Besides control steps within the engineering process, quality test controls such as ICP‐MS methods may help to characterize purchased raw materials, allowing a better risk assessment of their putative impact on the cell culture process.^46^, ^52^, ^54^ However, designing and developing new iron sources with low levels of impurities to decrease the risk of variability might be even more effective and must be considered.

## 5 CONCLUSION

This study describes the impact of iron raw material and its impurities on cell culture performance and recombinant protein glycosylation. While manganese was identified as the impurity in iron sources contributing to an overall improved cell performance, increasing levels of iron caused a decrease in cell growth, viability, and titer. Likewise, a contrary effect of iron and manganese was identified for the glycosylation profile of the tested recombinant proteins. Thus, this study highlights the need for low impurity iron sources in order to decouple the effects of iron and its impurities within cell culture experiments. If this is done appropriately, each element concentration can be controlled and adjusted individually, allowing the development of a consistent and stable cell culture process leading to reproducible quality attributes of the recombinant product. This may be particularly relevant within the field of biosimilars, whereby the drug substance release requires to match the product quality attributes within a very narrow range.

## AUTHOR CONTRIBUTIONS

**Christine H. Weiss:** investigation; validation; visualization; writing‐original draft. **Corinna Merkel:** supervision; validation. **Aline Zimmer:** supervision; validation.

## CONFLICT OF INTEREST

All authors are employees of Merck KGaA, Germany.

### PEER REVIEW

The peer review history for this article is available at https://publons.com/publon/10.1002/btpr.3148.

## Supporting information

**Figure S1** Supporting Information.

**Table S1** Impurity profile of iron source FC_Purch_.

## Data Availability

All data are contained within the manuscript, besides the Supplementary Table 1 and Supplementary Figure 1, which are provided separately.
