# Supplementary material for: Impact of iron raw materials and their impurities on CHO metabolism and recombinant protein product quality
Source: Biotechnol Prog. 2021 May 3;37(4):e3148. doi: 10.1002/btpr.3148 (PMC8459231; doi:10.1002/btpr.3148)
Supplement: Supplementary file 1 — Figure S1 Supporting Information. [file BTPR-37-e3148-s001.docx]

**
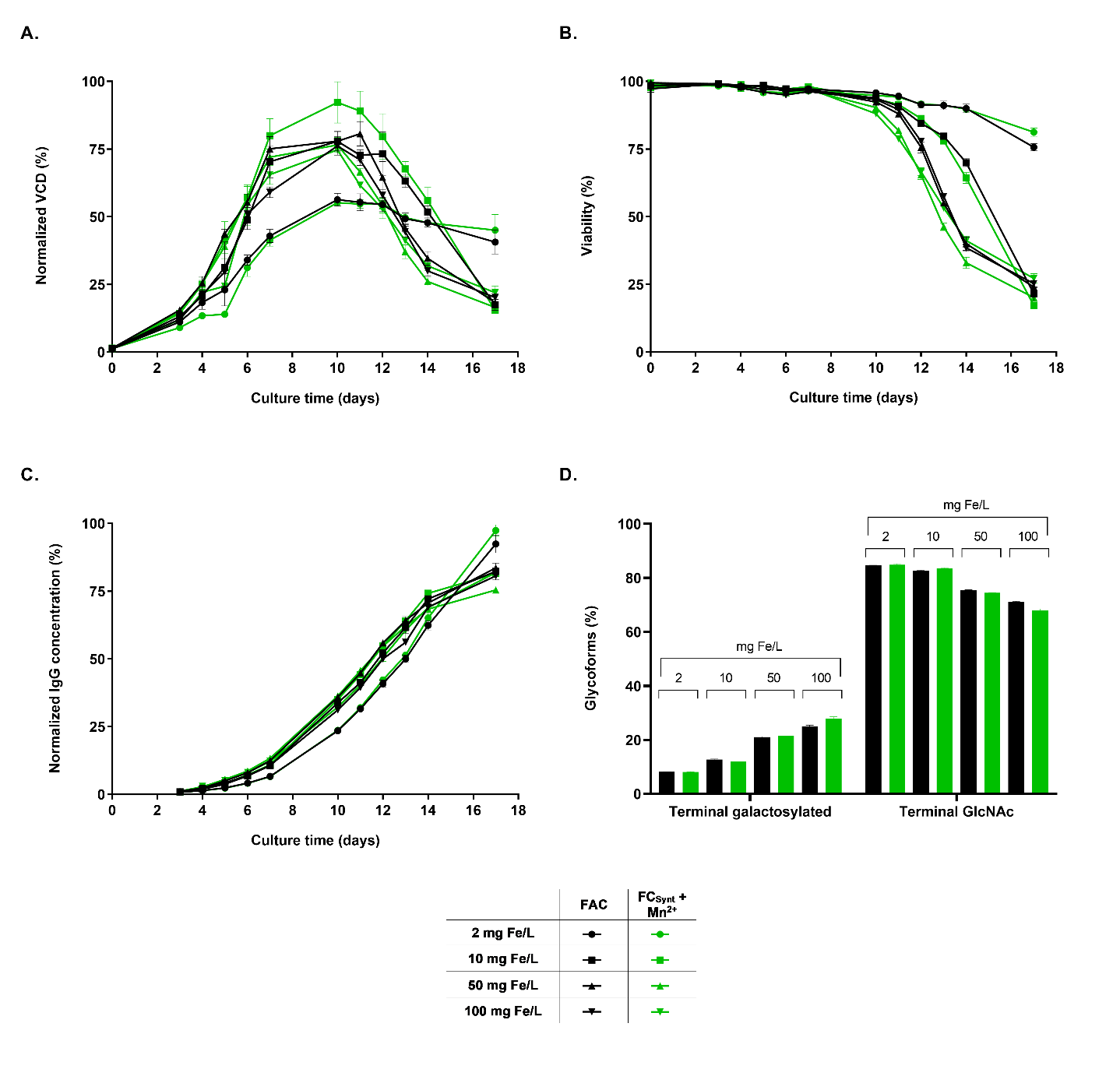
**

**Supplementary Figure 1.** Effect of iron sources FAC and FC_Synt_, supplemented with manganese, in CCM on cell performance of cell line 1 and glycosylation profile of mAb1. CHO K1 cells were cultivated in medium supplemented with either 2, 10, 50, or 100 mg Fe/L (FAC or FC_Synt_), whereas manganese was added to FC_Synt_ to achieve the same manganese concentration as present in FAC. N-glycosylation profile of mAb1 was determined on day 10 of fed-batch process. A. VCD in % normalized to the highest value. B. Viability in %. C. IgG concentration in % normalized to the highest value. D. N-glycosylation forms (terminal galactosylated and terminal GlcNAc) of mAb1 in %. Data are mean ± SD of four (A., B. and C.) or two (D.) biological replicates
