# Supplementary material for: Impact of iron raw materials and their impurities on CHO metabolism and recombinant protein product quality
Source: Biotechnol Prog. 2021 May 3;37(4):e3148. doi: 10.1002/btpr.3148 (PMC8459231; doi:10.1002/btpr.3148)
Supplement: Supplementary file 2 — Table S1 Impurity profile of iron source FCPurch. [file BTPR-37-e3148-s002.docx]

**Supplementary Table 1.** Impurity profile of iron source FC_Purch_. The impurity characterization was performed by ICP-MS, whereby the quantification was carried out by either a semiquantitative elemental screening method using a quadrupole-based ICP-MS or HR-ICP-MS utilizing an external calibration. B: boron, Mg: magnesium, Al: aluminum, K: potassium, Ca: calcium, Ti: titanium, V: vanadium, Cr: chromium, Mn: manganese, Co: cobalt, Ni: nickel, Cu: copper, Zn: zinc, Ga: gallium. All obtained calibration curves yielded a correlation coefficient of at least >0.999. * Values gained with HR-ICP-MS

| **µg/g** | **B** | **Mg** | **Al** | ***K** | ***Ca** | ***Ti** | ***V** | ***Cr** | **Mn** | **Co** | ***Ni** | **Cu** | **Zn** | **Ga** |
| --- | --- | --- | --- | --- | --- | --- | --- | --- | --- | --- | --- | --- | --- | --- |
| **FC_Purch_** | <1.0 | 4.0 | 56 | <10 | 95 | 4.0 | 2.4 | 80 | 530 | 4.0 | 34 | 3.4 | <25 | 1.1 |
